# Cellular rhinitis

**DOI:** 10.1186/2045-7022-5-S4-P43

**Published:** 2015-06-26

**Authors:** Alberto Macchi

**Affiliations:** 1 ORL Clinic University of Insubria, Varese, Italy

## 

Cellular Rhinitis represent a form of vasomotor rhinitis, they are studied by means of the nasal cytology which allows through the taking of a minimum amount of mucus in the nasal mucosa of studying with an optical microscope such cells. The cells are stained with May Grunwald Giemsa methods, such methods allows to highlight the major cellular components at the level of the surface layer of the nasal mucosa.

The forms of rhinitis cell are represented by non-allergic eosinophilic rhinitis (NAres), nonallergic rhinitis by mast cell eosinophilic (NARESMA), neutrophilic from non-allergic rhinitis (NARNE), nonallergic rhinitis by mast cell (Narma).

An epidemiological study in Italy, done by members dell’AICNA, Italian Academy of Nasal Citology) have highlighted these results on a population of 3872 people seen during a ENT visit: NARES 2.69%, 1.47% Naresma, 1.47% Narma, 16,50% allergic, 1.26% of narne, 2.38 overlapping, 70.53% normal, 1.47% yeast, 2.24 % bacterial.

Therefore represent a significant percentage of the vasomotor rhinitis.

The therapy today is not defined so many type of therapy are used by the clinician so the academy try to establish a rule to treat this rhinitis so actually are in study the treatment of such rhinitis with an association between a topical steroid, fluticasone propionate with a topical antihistamine azelastine hydrochloride. the primary results are satisfactory in the treatment of NARES.

